# Normal reference intervals for left atrial volume and cardiac dimensions according to age and sex assessed by two different methods using cardiac computed tomography angiography

**DOI:** 10.1093/ehjimp/qyaf125

**Published:** 2025-10-30

**Authors:** Benjamin Alos, Gwenaelle Oresve, Elsa Beard, Corinne Beaufort, Vincent Bergere, Thomas Roxburgh, Maxime Pin, Pierre-Jean Saulnier, Luc Christiaens, Claire Bouleti

**Affiliations:** Cardiology Department, University Hospital of Poitiers, Poitiers 86000, France; Clinical Investigation Center (INSERM 1402), University Hospital of Poitiers, Poitiers 86000, France; Radiology Department, Angouleme Hospital, Angouleme 16000, France; Cardiology Department, University Hospital of Poitiers, Poitiers 86000, France; Clinical Investigation Center (INSERM 1402), University Hospital of Poitiers, Poitiers 86000, France; Cardiology Department, University Hospital of Poitiers, Poitiers 86000, France; Cardiology Department, University Hospital of Poitiers, Poitiers 86000, France; Cardiology Department, University Hospital of Poitiers, Poitiers 86000, France; Cardiology Department, University Hospital of Poitiers, Poitiers 86000, France; Clinical Investigation Center (INSERM 1402), University Hospital of Poitiers, Poitiers 86000, France; Cardiology Department, University Hospital of Poitiers, Poitiers 86000, France; Cardiology Department, University Hospital of Poitiers, Poitiers 86000, France; Clinical Investigation Center (INSERM 1402), University Hospital of Poitiers, Poitiers 86000, France; FACT (French Alliance for Cardiovascular Trials) and ACTION Coeur, Paris 75000, France

**Keywords:** CCTA, left atrial volume, reference range, cardiac dimensions

## Abstract

**Aims:**

Left atrial volume (LAV) is a recognized prognostic marker of cardiovascular events. However, normal LAV thresholds on cardiac computed tomography angiography (CCTA) remain poorly defined, and the optimal assessment method is unclear. Our aim was to determine normal reference values of maximal systolic LAV (LAVmax) and ventricular dimensions normalized by age, sex, and body surface area (BSA) from CCTA by using dedicated semi-automated analysis software.

**Methods and results:**

This single-centre retrospective study included 250 healthy subjects with no cardiac history or significant CCTA abnormalities, stratified by sex and age. LAVmax was measured using both 3D and area–length methods, and all other cardiac chambers were analysed with dedicated 3D semi-automated software (Vitrea^®^). LAVmax increased significantly with age in both sexes but showed no sex difference when indexed to BSA. The 3D volumetric method was more reproducible (*r* = 0.86, *P* < 0.001) and yielded larger values compared with area–length method. Reference values for LAVmax and all cardiac chambers were provided for both sexes and each age group. Age, in concert with sex, was associated with significant differences in RV end-diastolic volume and LV ejection fraction (*P*-values 0.027 and 0.03).

**Conclusion:**

Indexed LAVmax was not significantly different across sexes but increased with age. LAVmax can be reliably reported from CCTA datasets, with 3D volumetric method providing the largest and most reproducible values. Normal values for all cardiac chambers according to age categories and sex were also provided. These normative values enhance the clinical utility of routine CCTA beyond coronary imaging.

## Graphical Abstract

**Graphical Abstract:**
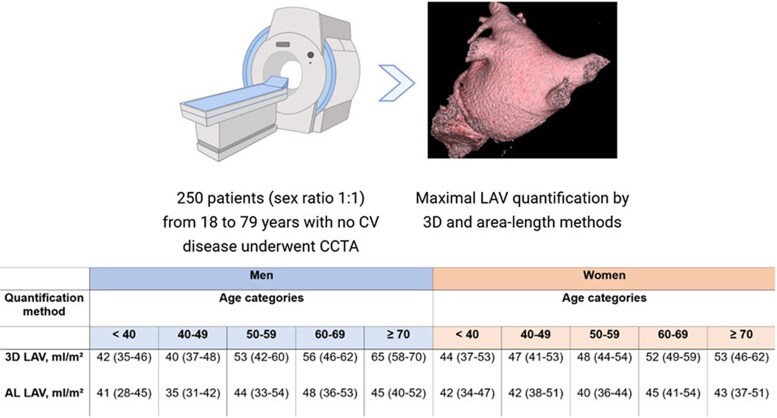

## Introduction

Left atrium (LA) enlargement is a frequent consequence of cardiac remodelling.^1^ It has already shown its prognostic value in most cardiac diseases, including valvular heart diseases and cardiomyopathies.^2^ Several studies have investigated normal left atrial volume (LAV) with 2D and 3D echocardiography and some with CMR, but data regarding cardiac computed tomography angiography (CCTA) reference intervals are scarce.

LAV is more accurate and a more robust marker of adverse events than 2 dimensional measurements (LA area or diameter). It varies with age, sex, and during the cardiac cycle with a maximal LAV (LAVmax) at end-systole (30–40% of the RR cycle) and a minimal LAV (LAVmin) at end-diastole (90–0% of the RR cycle), as measured with CT.^3^ A recent consensus statement proposed indexed LAV values using LAVmin, underestimating normal LAV.^1^ Reliable normal values of LAVmax are still lacking, with highly variable values reported in different series due to heterogeneity in the phase of the cycle, technique (volumetric or 2D), and the populations studied (mostly limited sample size of elderly patients with diseases associated with LA enlargement).^4^

CCTA has become an effective tool to evaluate numerous cardiac diseases, including coronary artery disease (CAD) or arrhythmias, e.g. before transcatheter intervention, with very large LA being associated with worse outcomes.^5–9^ Considering the growing indications of CCTA, its good spatial resolution and wide availability compared with CMR,^10^ providing reference intervals for cardiac dimensions is thus of paramount importance.^11^

The main aim of this study was to establish normal reference ranges for indexed LAVmax in healthy individuals across different age groups in both sexes. The secondary aim was to provide normative values for the dimensions of all other cardiac chambers.

## Methods

### Study design and population

All adult patients with no prior cardiac disease referred for CCTA between January 2014 and December 2016 at our institution were eligible for this study. CCTA examinations were mostly indicated according to international guidelines in patients with chest pain and low to intermediate pre-test probability, to rule out significant CAD.^12^ Exclusion criteria were: a past medical history of significant cardiac disease (obstructive CAD, arrhythmia, intra-cardiac device, any cardiomyopathy or significant valvular heart disease), poor quality of the images and/or coronary calcium score >1000, or any significant abnormal cardiac finding, including non-sinus rhythm during the examination. The study was conducted according to the ethical principles stated in the Declaration of Helsinki and in adherence with applicable guidelines for good clinical practice. Ethical approval was obtained from the local ethics committee (n°2025-04-02).

Our aim was to include adults with an equal number of participants of each sex across five age groups, ranging from 18 to over 80 years. Body surface area (BSA) was calculated according to the formula by Mosteller *et al*.^13^ For each patient, sex, age, height, weight, body mass index (BMI), BSA, blood pressure (BP), history of hypertension and presence of non-obstructive (<50% of coronary lumen diameter) CAD were recorded.

### Imaging protocol

All CCTA examinations were performed using a 64-slice CT scan (LightSpeed VCT, GE Healthcare, Milwaukee, WI, USA). Sublingual nitroglycerin was given to the patient as pre-medication if systolic BP was above 110 mmHg. Patients with no history of asthma were also given 2.5 to 7.5 mg of atenolol intravenously before CT if heart rate was above 70 bpm, to ensure a frequency below 60 bpm, as recommended.^14^

Acquisition of CCTA images was performed during a single breath-hold at end-inspiration, in the cranio-caudal direction, with retrospective electrocardiographic gating. Image acquisition was obtained using a triphasic injection (through an antecubital vein) of iodine-based contrast medium: 80 mL of an intravenous contrast agent (iomeprol 400 mg/mL, Iomeron^®^ 400, Bracco SA, Milan, Italy) injected at 4.5 mL/s followed by 40 mL (50%) injected at 4 mL/s and 30 mL of isotonic saline solution injected at 3 mL/s. Image acquisition triggering was set at an aortic attenuation density of 250 Hounsfield Units, with the region of interest placed at the descending aorta. Scanning parameters were as follows: tube rotation time of 350 ms, detector collimation of 64 × 0.625 mm, tube voltage and current selected according to patients’ body mass index between 100 and 120 kV, and between 280 and 500 mA, respectively, depending on patient weight. Ten data sets of non-overlapped transversal images were reconstructed retrospectively at every 10% of the R-R interval with slice thickness of 0.6 mm.

### Image analysis

**Figure 1:**
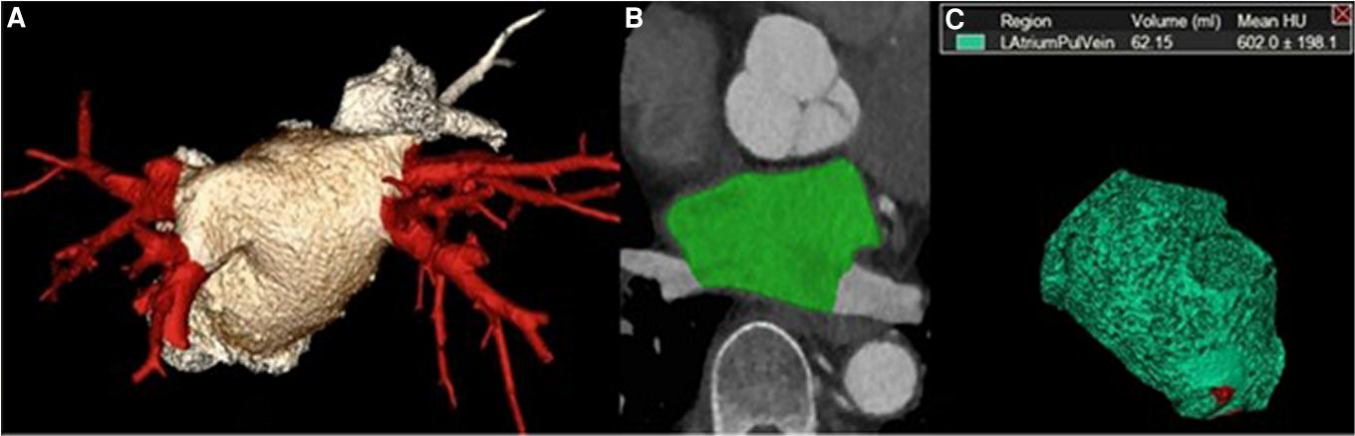
3D volume rendering (VR) reconstruction of the left atrium with PVs (in red), from a posterior view (*A*); 2D axial delineation of LA (green) with exclusion of PVs and LAA (*B*); 3D-VR reconstruction of the LAV without PVs and LAA, with calculated volume (*C*).

All examinations were analysed retrospectively using a dedicated workstation: DICOM data were recorded on McKesson^®^ PACS for morphological analysis, then exported to a semi-automated post-processing software (Vitrea^®^, Canon Medical Systems, Suresnes, France) for morphological analysis. LAV was assessed using two techniques, including a 3D method relying on automated segmentation of endocardial borders, reviewed and manually corrected by expert readers. We also performed a 2D-based evaluation of LAV using multi-planar reconstruction (MPR) with the manual area–length method (LAV = (0.85 × area(4ch) × area(2ch)/(mean atrial length from mid-mitral plane to the posterior LA wall)). Raw images consisted of contiguous axial slices, from which long-axis views were generated using MPR, analogous to standard echocardiographic projections. The LV long-axis—drawn from the mitral valve annulus to the apex—served as the reference for reconstructing the four-chamber (4CH) and two-chamber (2CH) views. The 4CH view included both mitral and tricuspid annuli; 2CH view was obtained by rotating the plane around the LV axis to visualize only LV and LA. In both cases, we quantified LAVmax (at LA end-diastole corresponding to 40% of RR cycle) by manually excluding PVs and LAAs, whose morphology and number have been recorded (*Figure 1*). To assess inter-observer variability, a double-blinded evaluation of LAV was conducted by two senior cardiac radiologists on 50 randomly selected scans, using both the 3D volumetric and AL methods.

Right atrial (RA) area was quantified from the ECG-gated CCTA dataset using MPR. From the previously reconstructed 4CH view, the endocardial contour of the RA was manually traced at end-systole. The contour included the RA chamber but excluded the right atrial appendage and the orifices of the superior and inferior vena cava, in accordance with echocardiographic and CT conventions. The delineated area was automatically calculated by the post-processing software and expressed in square centimetres (cm^2^).

Ventricular analysis followed the methodology described by Fuchs *et al*. (JCCT, 2012). RV and LV were assessed at end-systole and end-diastole using an external workstation (Vitrea 6.3, Vital Images, USA), with automated endocardial border detection. Boundaries were defined at the atrioventricular plane and great artery valves. Volumes were segmented based on attenuation differences between myocardium and contrast-enhanced blood, excluding papillary muscles. Chamber volumes were computed from voxel summation, and time–volume curves were automatically generated. Manual adjustments were made when needed. LV mass was calculated from corrected myocardial volume and multiplied by myocardial tissue density (1.055 g/mL).

### Statistical analysis

Quantitative variables are expressed as mean ± standard deviation or median (25th–75th percentiles) according to their distribution, and qualitative variables in numbers and percentages (%). Normality was tested by the Kolmogorov–Smirnov test and visually by distribution histograms and box-plots. Comparisons between 2 independent groups were made for quantitative variables by *t*-test or Mann–Whitney test as appropriate, and for qualitative variables by a χ^2^ test. Comparisons between the 5 independent groups were made using a Kruskal–Wallis test. Analyses of the determinants of LAV were performed by univariate and multi-variate analysis using multiple linear regression analysis. Correlation analyses were performed using a Pearson test. Inter-observer agreement on the 2 LAV measurement methods was assessed by calculating intra-class coefficient (ICC) using a two-way random ANOVA model. A *P* < 0.05 was considered statistically significant for the different analyses. All analyses were performed using SPSS version 23.0 (IBM Corp., Armonk, NY, USA).

## Results

### Baseline characteristics of the population

**Figure 2:**
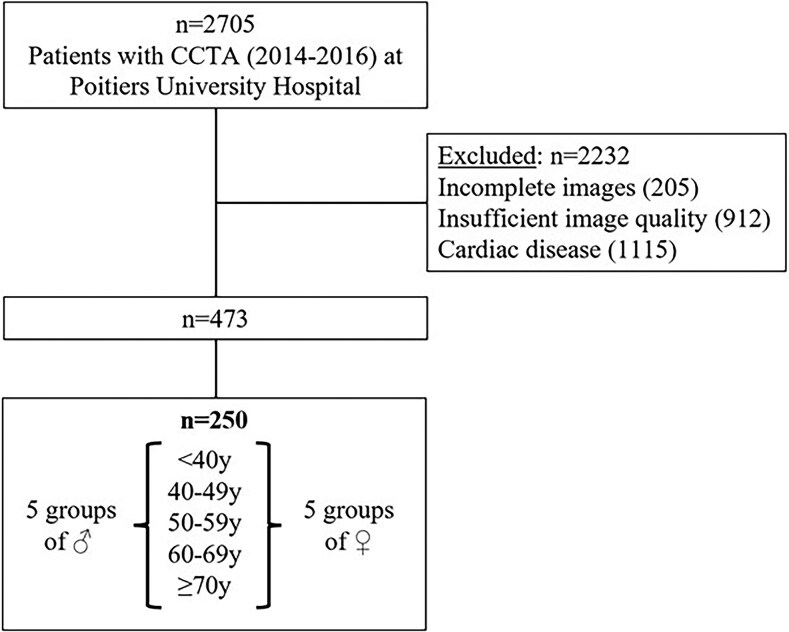
Flowchart of the study population.

Among the 2705 patients who underwent CCTA from January 2014 to December 2016, 2232 patients were excluded because of previous known cardiac disease (*n* = 1115), incomplete images or insufficient image quality for proper cardiac chambers measurements (*n* = 205 and 912, respectively). A total of 473 healthy individuals with complete CCTA were initially included. Among them, the smallest subgroup consisted of 25 women under 40 years of age. To ensure balanced representation, we then randomly selected 25 individuals from each of the remaining male and female age groups, resulting in 10 groups of 25 participants each, stratified by age and sex (*Figure 2*).

Demographic characteristics of patients are listed in *Table 1* for the whole population and according to sex. Mean age of the population was 54 ± 14 years and mean heart rate during CCTA was 63 ± 7 bpm. Height, weight, BSA, diastolic BP, and the presence of non-obstructive CAD were significantly different between men and women. There were 97 (39%) subjects with non-obstructive CAD, with significantly more men (46.4 vs. 31.2%, *P* < 0.02).

**Table 1:** Baseline characteristics according to gender

Parameters	Overall population (*n* = 250)	Men (*n* = 125)	Women (*n* = 125)	*P*-value
Age (years)	54 ± 14	54 ± 15	54 ± 14	0.79
Height (cm)	169 ± 10	175 ± 7	162 ± 7	**<0**.**001**
Weight (kg)	74 ± 16	81 ± 13	68 ± 15	**<0**.**001**
BMI (kg/m^2^)	26 ± 5	26 ± 4	26 ± 5	0.41
BSA (m^2^)	1.9 ± 0.2	2.0 ± 0.2	1.7 ± 0.2	**<0**.**001**
SBP (mmHg)	138 ± 21	139 ± 21 (n = 114)	137 ± 21 (n = 115)	0.61
DBP (mmHg)	77 ± 11	79 ± 10 (n = 114)	74 ± 11 (n = 115)	**<0**.**001**
HR (bpm)	63 ± 7	62 ± 7	63 ± 7	0.08
Hypertension	63 (25.3)	33 (26.6)	30 (24.0)	0.66
Non-obstructive CAD	97 (38.8)	58 (46.4)	39 (31.2)	**0**.**02**
Chest pain	21 (8.4)	10 (8.1)	11 (8.8)	1.0

Data are expressed as mean ± SD or n (%).

BMI, body mass index; BSA, body surface area; SBP, systolic blood pressure; CAD, coronary artery disease; DBP, diastolic blood pressure; HR, heart rate.

In both male and female populations, SBP, DBP, HR, the prevalence of hypertension, and non-obstructive CAD were significantly increased in older age groups (see Supplementary data online, *Table S1*).

### Atrial chambers dimensions and characteristics

The detailed analysis of atrial parameters showed no difference in the number of PVs or LAA shape between men and women, with cactus and chicken wing being the most prevalent (42 and 35%, respectively). Most patients had two left and two right PVs (89 and 78%, respectively), but a three right-PV form was frequent in both genders (20%), whereas a unique left venous pulmonary trunk was noted in 9% of patients. Regarding LA dimensions, neither area, nor volume were significantly different between men and women when indexed to BSA, with both 3D-volumetric and AL methods. On the other hand, RA area was significantly larger in men (2112 vs. 1966 mm^2^, *P* < 0.001) (*Table 2*).

**Table 2:** Atrial parameters in adults and according to sex

Parameters	All (*n* = 250)	Men (*n* = 125)	Women (*n* = 125)	*P*-value
CACS	47 ± 111	70 ± 142	25 ± 59	**0**.**001**
LAA shape				0.27
Chicken wing	88 (35)	46 (37)	42 (34)	
Windsock	30 (12)	19 (15)	11 (9)	
Cactus	105 (42)	46 (37)	59 (47)	
Cauliflower	27 (11)	14 (11)	13 (10)	
Number of right PVs				0.56
2	195 (78)	94 (75)	101 (81)	
3	50 (20)	28 (22)	22 (17.5)	
4	5 (2)	3 (2)	2 (1.5)	
Number of left PVs				0.37
1	23 (9)	14 (11)	9 (7)	
2	221 (88.5)	107 (86)	114 (91)	
3	6 (2.5)	4 (3)	2 (2)	
3D LAV (mL)	94 ± 27	102 ± 30	86 ± 20	**<0**.**001**
Indexed 3D LAV (mL/m^2^)	51 ± 13	52 ± 14.8	49.5 ± 10	0.15
Area–length LAV (mL)	79 ± 24	84 ± 26	75 ± 21	**0**.**002**
Indexed area–length LAV (mL/m^2^)	43 ± 12	43 ± 13	43 ± 10	0.80
4ch LA area (mm^2^)	2303 ± 474	2341 ± 504	2263 ± 440	0.19
4ch RA area (mm^2^)	1966 ± 540	2112 ± 645	1820 ± 354	**<0**.**001**

Data are expressed as mean ± SD or n (%).

CACS, coronary artery calcium score; LA, left atrium; LAA, left atrial appendage; LAV, left atrial volume; PV, pulmonary veins; RA, right atrium.

In both sexes, indexed LAV (3D and AL) and RA area increased significantly with age (*Table 3*). Volumes obtained using 3D-method was higher than those measured with the AL volumes (51 ± 13 mL/m^2^ vs. 43 ± 11 mL/m^2^ respectively, *P* < 0.001).

**Table 3:** Atrial parameters in adults across age groups

	Men						Women					
Parameters	Age categories					*P*-value	Age categories					*P*-value
	<40	40–49	50–59	60–69	≥70		<40	40–49	50–59	60–69	≥70	
CACS	0 (0–0)	0 (0–0)	0 (0–23)	25 (13–52)	69 (27–301)	**<0**.**001**	0	0	0 (0–13)	1 (0–24)	37 (0–135)	**<0**.**001**
3D LAV (mL)	76 (68–92)	82 (71–100)	107 (84–127)	110 (89–119)	127 (108–144)	**<0**.**001**	83 (66–101)	80 (68–91)	83 (80–92)	85 (79–123)	87 (74–107)	0.11
AL LAV (mL)	77 (54–88)	71 (61–85)	87 (68–112)	94 (71–103)	86 (76–100)	0.06	73 (62–88)	78 (64–88)	70 (60–75)	78 (65–98)	72 (60–90)	0.23
4ch LA area (cm^2^)	22 (19–25)	22 (20–24)	25 (20–29)	24 (21–29)	23 (20–26)	0.12	23 (19–28)	22 (20–25)	23 (20–24)	23 (21–29)	22 (19–26)	0.32
4ch RA area (cm^2^)	17 (15–19)	20 (16–21)	19 (17–25)	23 (19–25)	28 (23–32)	**<0**.**001**	19 (16–20)	17 (14–19)	18 (15–19)	20 (16–22)	20 (18–22)	**0**.**008**
Indexed 3D LAV (mL/m^2^)	42 (35–46)	40 (37–48)	53 (42–60)	56 (46–62)	65 (58–70)	**0**.**001**	44 (37–53)	47 (41–53)	48 (44–54)	52 (49–59)	53 (46–62)	**0**.**001**
Indexed AL LAV (mL/m^2^)	41 (28–45)	35 (31–42)	44 (33–54)	48 (36–53)	45 (40–52)	**0**.**03**	42 (34–47)	42 (38–51)	40 (36–44)	45 (41–54)	43 (37–51)	**0**.**03**

Data are expressed as median (upper-lower limits).

CACS, coronary artery calcium score; LA, left atrium; LAA, left atrial appendage; LAV, left atrial volume; PV, pulmonary veins; RA, right atrium.

### Correlations between LAV measurements methods

**Figure 3:**
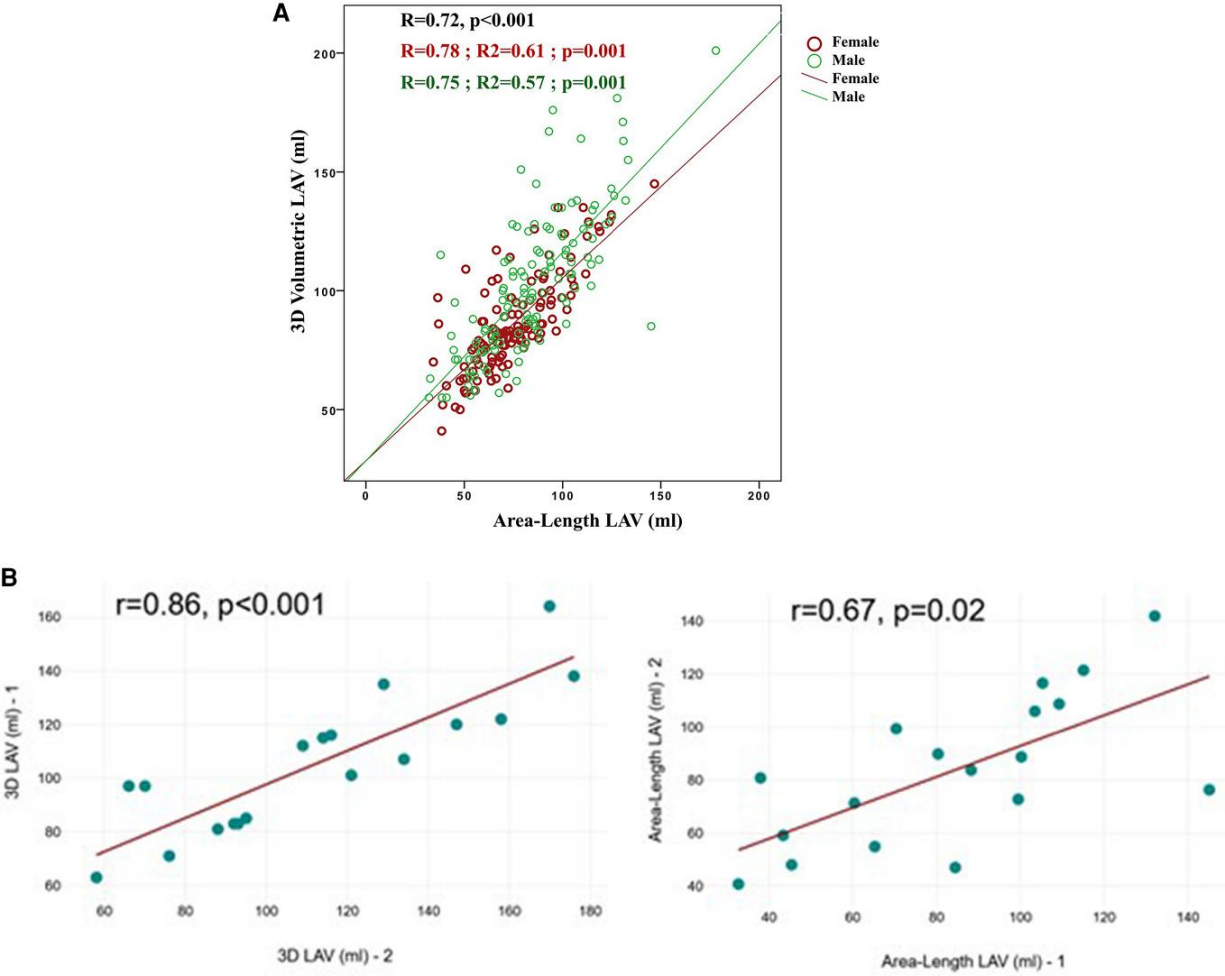
3D volumetric and area–length methods to assess LAV. (*A*) Correlations between 3D volumetric and area–length methods for LAV measurements. (*B*) Scatter diagram of inter-observer agreement of LAV using 3D volumetric and area–length methods.

The values of LAV obtained by the two methods were significantly correlated, in the general population (*R* = 0.72, *P* < 0.001) as well as in both sexes (*R* = 0.78 in women and 0.75 in men, *P* < 0.001) (*Figure 3A*).

However, the inter-observer variability assessed by ICC was better for 3D method (*r* = 0.86, *P* < 0.001), than AL (*r* = 0.67, *P* = 0.02) (*Figure 3B*).

### Determinants of LAV

In univariate analysis, in the global population, age, BSA, HR, LVEF, LV mass, and male sex were significantly associated with LAV by AL method (see Supplementary data online, *Table S2*), and in multi-variate analysis, only a higher age, higher BSA, lower LVEF and lower HR were independently associated with higher LAV (see Supplementary data online, *Table S3*)

Regarding 3D volumetric method, univariate analysis identified age, BSA, CCS, HR, LVEF, LV mass, male sex, and hypertension as factors significantly associated with LAV, and in multi-variate analysis, only a higher age, higher BSA, lower CCS, lower LVEF, and lower HR were independently associated with higher LAV (see Supplementary data online, *Tables S4* and *S5*). A second model including SBP and DBP instead of binary hypertension did not alter the results of the multi-variate analysis for any of the LAV assessment methods. Regardless of the method used, hypertension, LV mass and RVEF did not influence LAV.

### Ventricular dimensions and function

All absolute and indexed chamber dimensions were signiﬁcantly larger in men, except for LVESV/BSA with only borderline significance (*Table 4*). Enlargement being homogeneous, both LVEF and RVEF were comparable between sexes.

**Table 4:** Ventricular dimensions and function in adults and according to sex

Parameters	All (*n* = 250)	Men (*n* = 125)	Women (*n* = 125)	*P*-value
	Mean ± SD (LL-UL)	Mean ± SD (LL-UL)	Mean ± SD (LL-UL)	
LV parameters				
LVEF (%)	66 ± 7 (52–80)	66 ± 7 (69–73)	66 ± 7 (69–73)	0.90
LVEDV (mL)	139 ± 35 (69–209)	153 ± 36 (81–225)	125 ± 28 (69–181)	**<0**.**0001**
LVESV (mL)	48 ± 20 (8–88)	53 ± 22 (9–97)	44 ± 16 (12–76)	**<0**.**0001**
LVSV (mL)	90 ± 20 (50–130)	99 ± 20 (59–139)	82 ± 16 (50–114)	**<0**.**0001**
LVCO (mL)	5.6 ± 1.3 (3.0–8.2)	6.1 ± 1.2 (3.7–9.3)	5.1 ± 1.1 (2.9–7.3)	**<0**.**0001**
LVM (g)	109 ± 32 (45–173)	131 ± 28 (56–187)	88 ± 18 (52–124)	**<0**.**0001**
LVEDV/BSA (mL/m^2^)	74 ± 16 (42–106)	77 ± 18 (41–113)	71 ± 13 (45–97)	**0**.**002**
LVESV/BSA (mL/m^2^)	26 ± 9 (8–44)	27 ± 11 (5–49)	25 ± 8 (9–41)	0.06
LVSV/BSA (mL/m^2^)	48 ± 10 (28–68)	50 ± 11 (28–72)	46 ± 8 (30–62)	**0**.**003**
LVCI (mL/m^2^)	3.0 ± 1 (1–5)	3.1 ± 0.6 (1.9–4.3)	2.9 ± 0.6 (1.7–4.1)	**0**.**03**
LVM/BSA (g/m^2^)	58 ± 13 (32–84)	66 ± 12 (42–90)	50 ± 8 (34–66)	**<0**.**0001**
RV parameters				
RVEF (%)	54 ± 6 (42–66)	53 ± 6 (41–65)	54 ± 5 (44–64)	0.23
RVEDV (mL)	151 ± 38 (75–227)	172 ± 34 (104–240)	131 ± 29 (73–189)	**<0**.**0001**
RVESV (mL)	72 ± 23 (26–118)	85 ± 21 (43–127)	60 ± 16 (28–92)	**<0**.**0001**
RVSV (mL)	79 ± 18 (43–115)	88 ± 17 (54–122)	71 ± 15 (41–101)	**<0**.**0001**
RVCO (mL)	4.9 ± 1.2 (2.5–7.3)	5.4 ± 1.2 (3.0–7.8)	4.5 ± 1.0 (2.5–6.5)	**<0**.**0001**
RVEDV/BSA (mL/m^2^)	80 ± 17 (46–114)	87 ± 17 (53–121)	74 ± 13 (48–100)	**<0**.**0001**
RVESV/BSA (mL/m^2^)	38 ± 10 (18–58)	43 ± 10 (23–63)	34 ± 8 (18–50)	**<0**.**0001**
RVSV/BSA (mL/m^2^)	42 ± 9 (24–60)	44 ± 9 (26–62)	40 ± 7 (26–54)	**<0**.**0001**

Data are expressed as mean ± SD and upper-lower limits.

BSA, body surface area; LVCI, left ventricular cardiac index; LVCO, left ventricular cardiac output; LVEF, left ventricular ejection fraction; LVEDV, left ventricular end diastolic volume; LVESV, left ventricular end systolic volume; LVM, left ventricular mass; LVSV, left ventricular systolic volume; RVCI, right ventricular cardiac index; RVCO, right ventricular cardiac output; RVEF, right ventricular ejection fraction; RVEDV, right ventricular end diastolic volume; RVESV, right ventricular end systolic volume; RVSV, right ventricular systolic volume.

Due to differences in chambers dimensions, specific analysis according to age was conducted separately by sex.

In the male population, LV mass (LVM) and LV cardiac output (LVCO) (indexed to BSA and non-indexed) were the only parameters significantly different across groups, decreasing with age. Regarding the RV, RVEF was the only parameter significantly different across age groups (see Supplementary data online, *Table S6*).

In women, LV dimensions were almost all significantly different across age categories (with the notable exception of LVM), with decreasing values in older patients. However, LVEF was found to be lower in patients <40 years of age. On the other hand, RVEF and cardiac dimensions were not significantly different between age groups, except for RVEDV (indexed to BSA and non-indexed), significantly lower in older patients (see Supplementary data online, *Table S7*).

## Discussion

In this study, normal values for LAVmax were established, with a comparison between the two commonly used acquisition methods, across age groups and sexes, based on CCTA images from patients with no history of cardiac disease and no significant abnormalities on CT. This work also provides reference ranges for all cardiac chambers, aiming to fully leverage CCTA data in routine practice, beyond the sole visualization of the coronary arteries.

### Normal values of LAV

In this study, the mean LAVmax in the global population of 250 patients was 94 ± 27 mL (102 ± 30 mL in men, 86 ± 20 mL in women) and 51 ± 13 mL/m^2^ when indexed to BSA using the 3D method, without significant overall differences between men and women. Other published CT studies reported comparable dimensions, with LAVmax = 101.7 ± 27.2 mL in a 50 patients study by Abadi *et al*.,^15^ or indexed LAVmax of 50.7 ± 14 mL/m^2^, in an heterogeneous population of 150 patients, some with cardiac diseases.^4^ However, there was no subgroup in these studies to establish reference values according to age and sex. We observed larger LAV in women up to the age of 50, consistent with findings from a prior CMR study.^16^ This difference may be driven by hormonal factors, particularly higher oestrogen levels leading to increased pre-load and LA enlargement, with reversal after menopause. The only cohort with 3D volumetric analysis of healthy subjects divided in age groups was composed of 569 patients included in the Copenhagen General Population Study, but this population was predominantly female (67%) and the methods used to evaluate LAV were different than in our study: LAA was included as part of LA and images were acquired during ventricular diastole, representing minimal LAV, with volumes logically lower than our findings (81 ± 18 mL in men; 67 ± 14 mL in women).

In the 2019 European consensus paper,^17^ cardiac dimensions values using CMR in a global population of 478 patients from 20 to 80 years of age reported normal ranges for LAV in men between 47 and 107 mL (i.e. 26–52 mL/m^2^) and in women between 38 and 98 mL (27–53 mL/m^2^). These findings are consistent with our study. However, normal LAV values were not stratified by age, and LAV was not measured using the same method. In accordance with international imaging guidelines,^18^ PVs and the LAA were excluded from LAV delineation in our work, whereas the European CMR consensus included the LAA, potentially leading to LAV overestimation.

### Normal values for ventricular volumes and function

The LVEDV/VD and LVEDV/VD values reported in our series are extremely close to those published in a CMR meta-analysis,^19^ with a mean LVEDV of 153.4 ± 38.6 mL in men and 125 ± 32 mL in women (vs. 155 ± 30 mL and 123 ± 22 mL in CMR, respectively) and mean LVEDV of 180.4 ± 48.2 mL in men and 133 ± 32 mL in women (vs. 166 ± 39 mL and 122 ± 27 mL respectively on CMR), suggesting CCTA offers a close substitute to cardiac chambers evaluation with CMR being the reference. The decrease in LVM with age we observed in men could be linked to various factors, including reduced intense physical activity with aging, lower levels of BP and testosterone.^20^

### Anatomical variants of pulmonary veins

Our data concur with the literature, with 10 to 20% of variants with 1 left pulmonary venous common trunk (9% in our series) and 20 to 25% of forms with 3 right PVs (20% in our series).^21^ In addition, sex had no influence on the number of PVs, unlike other studies, where the prevalence of variants in the number of PVs was higher in women.^22^

### Limitations

This was a monocentre study, including mostly Caucasian patients, representative of the population in our region: our findings may thus not apply to other ethnic groups. Among the category of patients under 40 years, only a few subjects were younger than 30, with limited validity of our findings in young individuals. Moreover, the retrospective design of this study exposes to potential bias regarding missing or incomplete data from the medical records.

In addition, we included patients with hypertension, given that most patients referred to our institution for coronary CT had at least one cardiovascular risk factor apart from age, while others chose to evaluate only patients with no cardiovascular risk factors.^23^ However, in our work, hypertension did not influence LAV, irrespective of age or sex. This selection of patients does not therefore appear to limit the scope of our results.

## Conclusion

This work is the first to provide and compare two different methods—3D volumetric and AL—to establish normal values for LAVmax across five age classes in each sex. 3D method was the most reproducible technique, and provided the largest volumes compared with the AL method. Notably, indexed LAV increased with age but was not significantly different between sexes. These results highlight the reliability of a CCTA 3D volumetric approach to evaluate LAVmax, and encourage its use, especially in patients with suspected cardiac disease or undergoing cardiac procedures.

## Supplementary Material

qyaf125_Supplementary_Data

## Data Availability

The datasets used and/or analysed during the current study are available from the corresponding author on reasonable request.

## Lead author biography

Benjamin Alos joined the cardiology department of the University Hospital of Poitiers in 2018 as a senior physician. He is currently completing a PhD focusing on the multi-modality imaging in cardiac amyloidosis and aortic stenosis. His main topics of interest are cardiomyopathies, valvulart heart diseases and overall multi-modality imaging. He is currently serving as head of CMR unit and HCM programme.
