# Supplementary material for: Normal reference intervals for left atrial volume and cardiac dimensions according to age and sex assessed by two different methods using cardiac computed tomography angiography
Source: Eur Heart J Imaging Methods Pract. 2025 Oct 30;3(4):qyaf125. doi: 10.1093/ehjimp/qyaf125 (PMC12596262; doi:10.1093/ehjimp/qyaf125)
Supplement: qyaf125_Supplementary_Data [file qyaf125_supplementary_data.docx]

**Supplementary material**

**Supplementary table 1. Baseline characteristics according to age.**

|  | **Men (n=125)** | | | | | | **Women (n=125)** | | | | | |
| --- | --- | --- | --- | --- | --- | --- | --- | --- | --- | --- | --- | --- |
|  | **Age categories** | | | | | | **Age categories** | | | | | |
|  | **< 40**  **(n=25)** | **40-49**  **(n=25)** | **50-59**  **(n=25)** | **60-69**  **(n=25)** | **≥ 70**  **(n=25)** | **P-value** | **< 40**  **(n=25)** | **40-49**  **(n=25)** | **50-59**  **(n=25)** | **60-69**  **(n=25)** | **≥ 70**  **(n=25)** | **P-value** |
| Age | 35 (30-37) | 45 (43- 48) | 54 (52-57) | 63 (62-66) | 72 (71-75) | <0.001 | 36 (31-38) | 46 (43-48) | 54 (51-56) | 65 (62-67) | 73 (71-76) | <0.001 |
| BSA (m^2^) | 1.9 (1.8-2.1) | 2.0 (1.9-2.1) | 2.0 (1.9-2.2) | 1.9 (1.8-2.1) | 1.8 (1.8-2.1) | 0.02 | 1.9 (1.7-1.9) | 1.7 (1.6-1.87) | 1.7 (1.6-1.9) | 1.7 (1.6-1.9) | 1.7 (1.6-1.8) | 0.21 |
| SBP (mmHg) | 119 (114-126) (n=23) | 138 (115-143) (n=22) | 140 (130-158) (n=23) | 146 (135-151) (n=22) | 155 (140-170) (n=24) | <0.001 | 120 (110-127) | 140 (126-155) | 141 (122-156) | 141 (128-151) | 145 (132-160) | <0.001 |
| DBP (mmHg) | 70 (69-74) (n=23) | 79 (72-86) (n=22) | 80 (75-93) (n=23) | 79 (70-89) (n=22) | 87 (79-90) (n=24) | <0.001 | 70 (64-74) | 80 (71-88) | 75 (70-86) | 70 (65-81) | 68 (60-80) | 0.006 |
| HR (bpm) | 66 (60-70) | 63 (58-71) | 59 (57-63) | 59 (57-63) | 60 (55-65) | 0.004 | 60 (56-66) | 65 (58-69) | 65 (59-70) | 62 (59-64) | 62 (58-66) | 0.038 |
| Hypertension | 1 (4) | 3 (12) | 7 (28) | 10 (40) | 12 (50) | 0.001 | 3 (12) | 3 (12) | 5 (20) | 8 (32) | 11 (44) | 0.03 |
| Non obstructive CAD | 3 (12) | 10 (40) | 5 (20) | 20 (80) | 20 (80) | <0.001 | 1 (4) | 6 (24) | 6 (24) | 10 (40) | 16 (64) | <0.001 |
| Chest pain | 2 (8) | 1 (4) | 4 (16) | 2 (8) | 1 (4) | 0.53 | 2 (8) | 1 (4) | 4 (16) | 1 (4) | 3 (12) | 0.50 |

BSA, Body Surface Area; CAD, Coronary Artery Disease; DBP, Diastolic Blood Pressure; HR: Heart Rate; SBP, Systolic Blood Pressure

**Supplementary Table 2. Univariate analysis of the determinants of LAV by area-length method.**

| **Variables** | **Beta Coefficient (β)** | **95% CI** | **P-value** |
| --- | --- | --- | --- |
| **Age (years)** | 0.23 | 0.02 ; 0.43 | 0.03 |
| **BSA (m^2^)** | 41.52 | 29.67 ; 53.38 | <0.001 |
| **HR (BPM)** | -0.55 | -0.97 ; -0.13 | 0.010 |
| **CCS** | -0.003 | -0.03 ; 0.02 | 0.81 |
| **LVEF (%)** | -0.78 | -1.17 ; -0.38 | <0.001 |
| **LVM (g)** | 0.22 | 0.14 ; 0.31 | <0.001 |
| **RVEF (%)** | -0.33 | -0.79 ; 0.13 | 0.16 |
| **Male sex** | 9.08 | 3.26 ; 14.91 | 0.002 |
| **Hypertension** | 2.40 | -4.44 ; 9.23 | 0.49 |

BSA: Body Surface Area; CCS: Coronary Calcium Score; HR: Heart Rate; LVEF: Left Ventricular Ejection Fraction; LVM: Left Ventricular Mass; RVEF: Right Ventricular Ejection Fraction.

**Supplementary Table 3. Multivariate analysis of the determinants of LAV by area-length method.**

| **Parameters** | **Beta Coefficient (β)** | **95% CI** | **P-value** |
| --- | --- | --- | --- |
| **Age (years)** | 0.38 | 0.16 ; 0.52 | <0.001 |
| **BSA (m²)** | 41.1 | 29.82 ; 52.28 | <0.001 |
| **HR (bpm)** | -0.50 | -0.87 ; -0.13 | 0.009 |
| **LVEF (%)** | -0.84 | -1.20 ; -0.48 | <0.001 |

BSA: Body Surface Area; HR: Heart Rate; LVEF: Left Ventricular Ejection Fraction.

**Supplementary Table 4. Univariate analysis of the determinants of LAV by 3D volumetric method.**

| **Parameters** | **Beta Coefficient (β)** | **95% CI** | **P-value** |
| --- | --- | --- | --- |
| **Age (years)** | 0.71 | 0.50 ; 0.93 | **<0.001** |
| **BSA (m²)** | 51.45 | 38.38 ; 64.53 | **<0.001** |
| **CCS** | 0.052 | 0.02 ; 0.08 | 0.001 |
| **HR (bpm)** | -0.90 | -1.37 ; -0.44 | **<0.001** |
| **LVEF (%)** | -0.87 | -1.31 ; -0.42 | **<0.001** |
| **LVM (g)** | 0.31 | 0.21 ; 0.41 | **<0.001** |
| **RVEF (%)** | -0.32 | -0.83 ; -0.20 | 0.23 |
| **Male sex** | 15.99 | 9.62 ; 22.37 | **<0.001** |
| **Hypertension** | 11.11 | 3.54 ; 18.69 | 0.004 |

BSA: Body Surface Area; CCS: Coronary Calcium Score; HR: Heart Rate; LVEF: Left Ventricular Ejection Fraction; LVM: Left Ventricular Mass; RVEF: Right Ventricular Ejection Fraction.

**Supplementary Table 5. Multivariate analysis of the determinants of LAV by 3D volumetric method.**

| **Parameters** | **Beta Coefficient (β)** | **95% CI** | **P** |
| --- | --- | --- | --- |
| Age (years) | 0.46 | 0.26 ; 0.67 | <0.001 |
| BSA (m^2^) | 37.85 | 22.66 ; 53.03 | <0.001 |
| CCS | -0.03 | -0.05 ; -0.003 | 0.030 |
| HR (bpm) | -0.47 | -0.85 ; -0.08 | 0.017 |
| LVEF (%) | -0.85 | -1.20 ; -0.49 | <0.001 |

BSA: Body Surface Area; CCS: Coronary Calcium Score; HR: Heart Rate; LVEF: Left Ventricular Ejection Fraction.

**Supplementary table 6. Ventricular dimensions & function across age groups in men.**

| Men | | | | | | |
| --- | --- | --- | --- | --- | --- | --- |
|  | **Age categories** | | | | | **P-value** |
|  | **< 40** | **40-49** | **50-59** | **60-69** | **≥ 70** |  |
| LV parameters |  |  |  |  |  |  |
| LVEF (%) | 65 (60-68) | 65 (61-70) | 68 (59-71) | 68 (61-72) | 67 (62-70) | 0.38 |
| LVEDV (ml) | 157 (142-188) | 161 (133-184) | 146 (131-162) | 143 (126-162) | 147 (129-161) | 0.715 |
| LVESV (ml) | 61 (44-67) | 56 (35-69) | 49 (43-62) | 43 (36-60) | 46 (38-61) | 0.184 |
| LVSV (ml) | 101 (90-118) | 100 (90-110) | 98 (81-104) | 92 (87-108) | 94 (84-109) | 0.934 |
| LVCO (l/min) | 6.3 (5.9-7.3) | 6.6 (5.9-7) | 5.6 (4.8-6.7) | 5.6 (4.9-6.6) | 5.5 (4.8-6.4) | **0.033** |
| LVM (g) | 135 (118-145) | 121 (117-140) | 154 (138-168) | 118 (113-137) | 116 (100-126) | **<0.0001** |
| LVEDV/BSA (ml/m²) | 85 (69-99) | 80 (69-85) | 73 (60-84) | 74 (63-85) | 72 (69-83) | 0.057 |
| LVESV/BSA (ml/m²) | 29 (23-38) | 27 (17-36) | 25 (21-30) | 23 (17-29) | 24 (21-30) | 0.057 |
| LVSV/BSA (ml/m²) | 55 (43-60) | 49 (46-53) | 46 (40-55) | 49 (43-58) | 49 (45-57) | 0.546 |
| LVCI (l/min/m²) | 3.4 (3.0-4.0) | 3.2 (2.9-3.5) | 2.8 (2.4-3.3) | 2.8 (2.6-3.4) | 3.0 (2.5-3.2) | **0.020** |
| LVMI (g/m²) | 68 (62-75) | 62 (57-71) | 74 (68-85) | 64 (60-68) | 60 (51-65) | **0.001** |
| RV parameters |  |  |  |  |  |  |
| RVEF (%) | 50 (48-54) | 49 (46-53) | 59 (49-68) | 53 (48-55) | 53 (49-55) | **0.016** |
| RVEDV (ml) | 165 (155-195) | 182 (146-198) | 189 (139-206) | 166 (134-188) | 180 (159-189) | 0.886 |
| RVESV (ml) | 81 (74-97) | 86 (74-100) | 96 (70-113) | 76 (63-90) | 87 (75-96) | 0.384 |
| RVCO (ml) | 85 (74-98) | 88 (73-95) | 88 (77-98) | 89 (69-101) | 87 (77-99) | 0.896 |
| RVCO (l/min) | 5.7 (4.8-6.5) | 5.3 (4.5-6.5) | 5.1 (4.4-6.2) | 5.3 (4.4-5.9) | 5.2 (4.7-5.9) | 0.972 |
| RVEDV/BSA (ml/m²) | 86 (78-96) | 88 (76-95) | 87 (78-101) | 87 (66-97) | 87 (83-97) | 0.972 |
| RVESV/BSA (ml/m²) | 43 (39-50) | 43 (36-49) | 42 (36-53) | 40 (34-48) | 42 (39-48) | 0.934 |
| RVSV/BSA (ml/m²) | 44 (39-53 | 43 (38-45) | 44 (37-49) | 45 (37-50) | 45 (41-51) | 0.600 |
| RVCI (l/min/m²) | 2.8 (2.5-3.5) | 2.6 (2.3-3.0) | 2.4 (2.1-3.1) | 2.7 (2.0-3.0) | 2.7 (2.3-3.2) | 0.774 |

BSA, Body Surface Area; LVCI, Left Ventricular Cardiac Index; LVCO, Left Ventricular Cardiac Output; LVEF, Left Ventricular Ejection Fraction; LVEDV, Left Ventricular End Diastolic Volume; LVESV, Left Ventricular End Systolic Voilume; LVM, Left Ventricular Mass; LVSV, Left Ventricular Systolic Volume; RVCI, Right Ventricular Cardiac Index; RVCO, Right Ventricular Cardiac Output; RVEF, Right Ventricular Ejection Fraction; RVEDV, Right Ventricular End Diastolic Volume; RVESV, Right Ventricular End Systolic Volume; RVSV, Right Ventricular Systolic Volume

**Supplementary table 7. Ventricular dimensions and function across age groups in women.**

| **Women** | | | | | | |
| --- | --- | --- | --- | --- | --- | --- |
|  | **< 40** | **40-49** | **50-59** | **60-69** | **≥ 70** | **P-value** |
| LV parameters |  |  |  |  |  |  |
| LVEF (%) | 61 (58-67) | 66 (63-69) | 68 (62-71) | 66 (63-70) | 68 (62-74) | **0.035** |
| LVEDV (ml) | 134 (120-167) | 130 (112-146) | 117 (113-120) | 122 (106-146) | 111 (86-118) | **<0.0001** |
| LVESV (ml) | 49 (42-65) | 43 (37-50) | 38 (31-46) | 45 (28-58) | 33 (25-42) | **0.004** |
| LVSV (ml) | 84 (79-107) | 86 (77-94) | 80 (74-84) | 81 (72-87) | 72 (60-87) | 0.107 |
| LVCO (l/min) | 5.6 (4.6-6.0) | 5.4 (4.7-6.6) | 5.1 (4.7-5.5) | 5.1 (4.5-5.5) | 4.6 (3.8-5.3) | 0.234 |
| LVM (g) | 88 (79-102) | 85 (81-92) | 89 (82-97) | 89 (75-94) | 79 (75-99) | 0.611 |
| LVEDV/BSA (ml/m²) | 73 (68-80) | 73 (67-81) | 66 (60-75) | 72 (61-80) | 62 (54-70) | **0.002** |
| LVESV/BSA (ml/m²) | 27 (23-34) | 24 (22-30) | 21 (19-24) | 26 (18-31) | 19 (14-25) | **0.026** |
| LVSV/BSA (ml/m²) | 48 (42-54) | 49 (45-52) | 45 (40-49) | 46 (43-48) | 42 (38-48) | 0.096 |
| LVCI (l/min/m²) | 3.0 (2.5-3.4) | 3.2 (2.8-3.4) | 2.8 (2.6-3.1) | 2.9 (2.6-3.2) | 2.8 (2.2-3.0) | 0.257 |
| LVMI (g/m²) | 51 (45-54) | 48 (46-55) | 50 (47-54) | 48 (45-53) | 47 (44-53) | 0.60 |
| RV parameters |  |  |  |  |  |  |
| RVEF (%) | 53 (48-56) | 53 (52-58) | 52 (48-56) | 54 (50-58) | 55 (52-57) | 0.368 |
| RVEDV (ml) | 149 (131-170) | 133 (124-146) | 124 (120-132) | 128 (110-146) | 112 (90-124) | **<0.0001** |
| RVESV (ml) | 71 (59-84) | 59 (54-70) | 58 (53-67) | 56 (45-69) | 48 (42-60) | 0.076 |
| RVCO (ml) | 76 (64-90) | 71 (68-81) | 66 (64-72) | 68 (62-85) | 61 (54-69) | **0.002** |
| RVCO (l/min) | 4.6 (4.0-5.5) | 4.6 (4.1-5.6) | 4.3 (4.0-4.8) | 4.2 (3.9-5.4) | 3.9 (3.1-4.4) | **0.003** |
| RVEDV/BSA (ml/m²) | 80 (76-89) | 76 (70-87) | 72 (63-80) | 73 (67-82) | 65 (57-78) | **0.006** |
| RVESV/BSA (ml/m²) | 39 (34-43) | 36 (31-39) | 34 (30-37) | 35 (26-37) | 28 (24-37) | 0.075 |
| RVSV/BSA (ml/m²) | 42 (40-46) | 41 (39-43) | 39 (33-42) | 42 (37-44) | 37 (32-41) | **0.029** |
| RVCI (l/min/m²) | 2.6 (2.3-2.9) | 2.7 (2.2-3.0) | 2.4 (2.2-2.8) | 2.5 (2.3-2.7) | 2.2 (1.9-2.6) | 0.203 |

BSA, Body Surface Area; LVCI, Left Ventricular Cardiac Index; LVCO, Left Ventricular Cardiac Output; LVEF, Left Ventricular Ejection Fraction; LVEDV, Left Ventricular End Diastolic Volume; LVESV, Left Ventricular End Systolic Voilume; LVM, Left Ventricular Mass; LVSV, Left Ventricular Systolic Volume; RVCI, Right Ventricular Cardiac Index; RVCO, Right Ventricular Cardiac Output; RVEF, Right Ventricular Ejection Fraction; RVEDV, Right Ventricular End Diastolic Volume; RVESV, Right Ventricular End Systolic Volume; RVSV, Right Ventricular Systolic Volume
